# Transgastric migration of gossypiboma remedied with endoscopic removal: a case report

**DOI:** 10.1186/1756-0500-6-413

**Published:** 2013-10-14

**Authors:** Alper Sozutek, Serdar Yormaz, Hakan Kupeli, Burhan Saban

**Affiliations:** 1 Department of Gastroenterological Surgery, Necip Fazil State Hospital, Kahramanmaras, Turkey; 2 Department of Gastroenterological Surgery, Kahramanmaras Necip Fazil State Hospital, Kahramanmaras, Turkey

**Keywords:** Gossypiboma, Intraluminal migration, Retained surgical sponge, Gastroscopy

## Abstract

**Background:**

Retained surgical instrument or sponge following an intra-abdominal surgery is a potentially dangerous medico-legal problem. The condition may manifest either as asymptomatic or severe gastrointestinal complications. Transmural migration of gossypiboma is a rare entity that may lead to bowel or visceral perforation, obstruction and/or fistula formation. Transmural migration of an intra-abdominal gossypiboma has been reported to occur in stomach, ileum, colon, bladder, vagina and diaphragm. To our knowledge, this is the fifth case reported in the medical literature. However, we report the first case of the largest gossypiboma to date: a surgical gas compress measuring 20 × 20 cm which was successfully treated endoscopically.

**Case presentation:**

A 52-year-old woman with obstructive jaundice was referred to our clinic. She had a medical history of cholecystectomy and T-tube drainage for choledocholithiasis a year previously. Abdominal ultrasonography and computed tomography revealed a mass located into the stomach which was compatible with gastric carcinoma. On the gastroscopy, a surgical gas compress that had totally migrated into the stomach was observed. The compress was successfully removed by gastroscopy through the esophagus. The recovery of the patient was uneventful.

**Conclusion:**

Transmural migration of gossypiboma into the stomach should be considered in the differential diagnosis of any postoperative patient with obstructive jaundice symptoms. Endoscopy may be feasible for both diagnosis and treatment even though the size of gossypiboma is large. However, surgery should be considered in case of fixed reaction or incomplete migration of gossypiboma located into the stomach.

## Background

Gossypiboma is the term used to describe a retained non-absorbable surgical material that is composed of cotton matrix which leads to serious surgical complications for both patient and surgeon [1]. The incidence is unclear due to medico-legal importance of the entity. Clinical symptoms related to intra-abdominal gossypiboma may vary from mild abdominal pain to major complications including bowel or visceral perforation, obstruction, fistula formation or sepsis [2]. Despite its rarity, transmural migration of gossypiboma is one of the possible causes of these gastrointestinal complications. Transmural migration of an intra-abdominal gossypiboma has been reported to occur in stomach, ileum, colon, bladder, vagina and diaphragm [3]. To our knowledge, this is the fifth reported case of transgastric migration of a gossypiboma in the medical literature. However, all of them were a standard size of surgical sponge and three of them were removed endoscopically [3,4]. Herein we report the largest gastric gossypiboma to date which was first diagnosed and then successfully treated endoscopically.

## Case presentation

**Figure 1:**
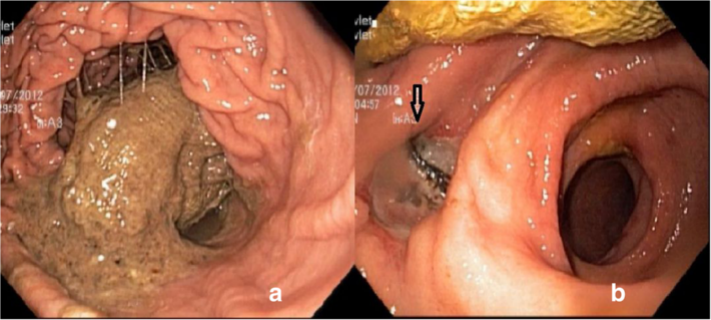
**Endoscopic view of surgical gas compress a) surgical compress filled two-thirds of the stomach b) view of antrum after removal of swab.** (Migration side is marked with arrow).

**Figure 2:**
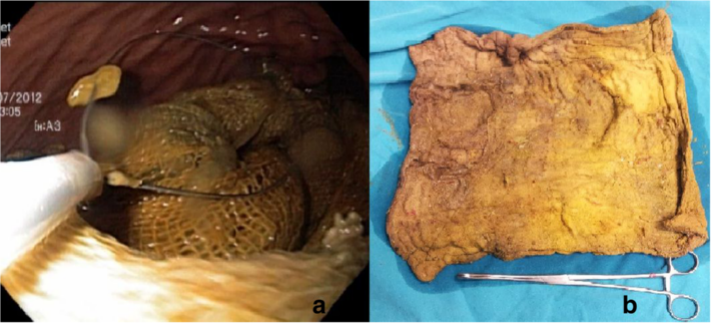
Endoscopic removal of surgical gas compress a) removing the swab by snare b) view of surgical swab after the procedure.

A 52-year-old woman presenting with a two-week history of epigastric pain and vomiting was referred to our clinic. She had a medical history of cholecystectomy and T-tube drainage operation for choledocholithiasis a year previously. Abdominal pain was of recent onset and mainly in the upper quadrants of her abdomen. Laboratory parameters revealed high levels of leukocyte (13,1×10^3^μL) and cholestatic enzymes; aspartate aminotransferase (AST): 51 U/L, alanine aminotransferase (ALT): 37 U/L, alkaline phospatase (ALP): 143 U/L, gamma glutamyl transferase (GGT): 120 U/L and amylase: 220 U/L. Plain abdominal radiography and ultrasonography (USG) were unremarkable. The preliminary diagnosis was acute pancreatitis until abdominal computed tomography (CT) revealed a 10×8 cm mass located in the stomach which was compatible with a gastric carcinoma. However, we suspected a gossypiboma in the differential diagnosis due to the medical history of patient. Gastroscopy was performed for diagnosis and treatment. A written informed consent including surgical risks was obtained from the patient. The procedure was performed under sedation in the operation room with regards to possible urgent surgical intervention. On the gastroscopy, a large surgical gas compress which totally migrated and filled two thirds of the stomach was observed (Figure 1). The distal side of the compress was located in the bulbus. The surgical compress was loosened up with saline. Subsequently, it was grasped with saw-tooth forceps and pulled into the stomach. The compress was grasped again with a snare followed by releasing from the bulbus, then pulled out through the esophagus to the posterior region of the tongue. The material was then removed with gentle round motions from the mouth (Figure 2). No bleeding, fistula or injury was observed. All laboratory parameters were normal after one day following the procedure. The recovery of the patient was uneventful; and she was discharged 4 days following the procedure.

## Discussion

Retained surgical instrument or sponge following an intra-abdominal surgery is a potentially dangerous medico-legal problem. Despite a published incidence of 1:1000 to 1:1500 after intra-abdominal surgeries, it is encountered more commonly than reported [5]. The fear of litigation, disclosing the error by other clinicians or asymptomatic gossypiboma may mask the real incidence.

Gossypiboma induces two types of foreign body reactions; the first type is an aseptic fibrinous response that creates adhesions and encapsulation while the second type is an exudative reaction which leads to inflammatory reaction with abscess formation [6,7]. Clinical symptoms usually depend on the type of tissue reaction. Although the first type reaction causes mild clinical symptoms like a painless abdominal mass, even asymptomatic, exudative reactions may manifest as a severe clinical course resulting in intestinal perforation, obstruction, fistula formation or sepsis [1-8]. Migration of a retained sponge is a rare condition compared to abscess formation. It is a result of bodily response to extrude the foreign material by developing a fistula externally or into a hollow viscus. Transmural migration occurs as a result of inflammation in the intestinal wall that evolves to necrosis [4,8,9]. The migration site closes after complete migration of the surgical towel. The small intestine is the most affected site due to its thin wall and large outer surface. Compared with the intestines, the stomach is an unusual site for transmural migration due to its higher localization and thick wall [6,9]. Until now, this condition has been previously reported only in five cases [3,4,6,9]. Interestingly, all of them occurred after acute open cholecystectomy operations. Hence, we emphasize that acute cholecystectomy is a major factor that leads to this kind of complication.

Imaging procedures such as plain X-ray, USG, CT and/or magnetic resonance (MR) may usually be helpful for diagnosis. Basically, a “whorl-like” mass imaging on plain X-ray is usually enough for diagnosis. In addition, imaging of a hyperechogenic mass with hypoechoic rim on USG or a rounded mass with a dense central part and enhancing wall on CT are the basic signs of gossypiboma [5,10]. However, all can be inconclusive if the sponge does not have any radiological marker. Moreover, it is frequently misdiagnosed as intra-abdominal hematoma, abscess or neoplasm which leads to unnecessary radical surgical interventions. Hence, radiologic findings may not be reliable to rule out other pathologies as in our case. For this reason, gossypiboma should be considered in the differential diagnosis of any postoperative patient who presents with such suspicious radiological findings.

Gossypiboma should be removed as soon as possible to avoid further surgical complications and legal problems [1]. Although open surgery is the most common approach in the treatment of gossypiboma, according to the localization of gossypiboma and skills of the clinician, removal can be easily performed by minimally invasive techniques such as endoscopy or laparoscopy [1,4,6]. Although successful removals of surgical sponges by endoscopy has been reported before, the feasibility of endoscopy in removal of such a large surgical gas compress was unclear. To our knowledge, herein we report the first case of the largest gossypiboma published to date successfully treated endoscopically. Hence, we emphasize that endoscopy may be a good option in the removal of such a large gas compress located in the stomach. However, surgery should be considered when fixed reaction and/or partial migration has occurred.

It is notable that prevention should be discussed instead of treatment modalities. Patients undergoing emergency surgery, those with high body mass index, lengthy operations, inexperienced staff or unexpected change in surgical procedure are major risk factors for retained surgical materials [1,8]. Simple precautions like educating the staff, tagging the sponges with markers or peroperative multiple counts of sponges and materials should reduce the incidence of gossypiboma [8]. In addition, new technologies like electronic tagging of sponges may be helpful in decreasing the incidence [11]. However, the feasibility of the procedure for our country is questionable.

## Conclusion

Transmural migration of gossypiboma into the stomach should be considered in the differential diagnosis of any postoperative patient with obstructive jaundice symptoms. Endoscopy may be feasible for both diagnosis and treatment even though the size of gossypiboma is large. However, surgery should be considered in case of fixed reaction or incomplete migration of gossypiboma located into the stomach.

## Consent

Written informed consent was obtained from the patient for publication of this Case Report and any accompanying images. A copy of the written consent is available for review by the Editor-in-Chief of this journal.

## Abbreviations

AST: Aspartate aminotransferase; ALT: Alanine aminotransferase; ALP: Alkaline phospatase; GGT: Gamma glutamyl transferase; USG: Ultrasonography; CT: Computed tomography; MR: Magnetic resonance.

## Competing interests

The authors declare that they have no competing interests.

## Authors’ contributions

AS, SY participated in acquisition of data and drafting the manuscript. HK, BS participated in revising critically the manuscript and giving the final approval of the version to be published. All authors read and approved the final manuscript.
